# Supplementary material for: Auranofin targets UBA1 and enhances UBA1 activity by facilitating ubiquitin trans-thioesterification to E2 ubiquitin-conjugating enzymes
Source: Nat Commun. 2023 Aug 9;14:4798. doi: 10.1038/s41467-023-40537-x (PMC10412574; doi:10.1038/s41467-023-40537-x)
Supplement: Supplementary file 1 — Supplementary Information [file 41467_2023_40537_MOESM1_ESM.pdf]

Auranofin targets UBA1 and enhances UBA1 activity by facilitating ubiquitin trans-thioesterification to E2 ubiquitin-conjugating enzymes

Yan W et al

Supplemental information

Supplementary Figures 1-8
Supplementary Tables 1-2

Suppl. Fig. 1

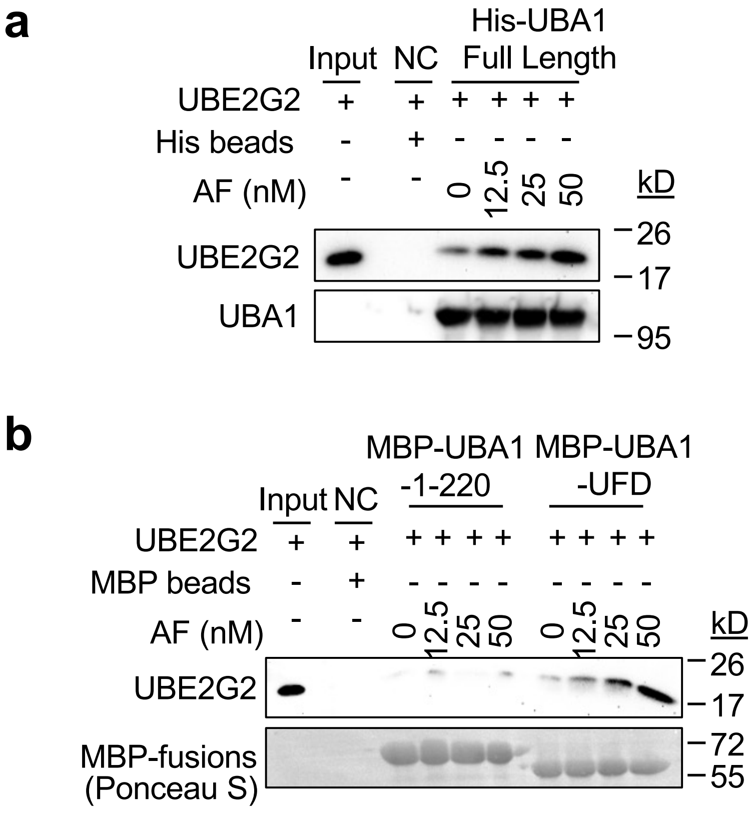

Suppl. Fig. 1: ***In vitro*** **pull-down assay testing the interaction of UBA1's UFD with UBE2G2**. **(a)** Effects of AF on 6His-UBA1 pulldown of UBE2G2 *in vitro*. **(b)** Effects of AF on MBP-UFD and MBP-N-terminal amino acids 1-220 pulldown of UBE2G2 *in vitro*. 6His-UBA1 and MBP-UBA1 fragment (amino acids 1-220 or UFD) immobilized on beads were pretreated with indicated concentrations of AF for 1.5 h at room temperature. After washed with lysis buffer at 5000 g for 3 times, purified His-UBE2G2 was added to each reaction, followed by incubation for 2 h at room temperature. After washing, 6His-UBA1 or MBP-UBA1 fragment-associated UBE2G2 detected by immunoblotting. Beads alone treated as negative control (NC). The 6His-UBA1 was detected by immunoblotting and the MBP-UBA1 proteins were stained with Ponceau S. Source data are provided as a Source Data file.

Suppl. Fig. 2

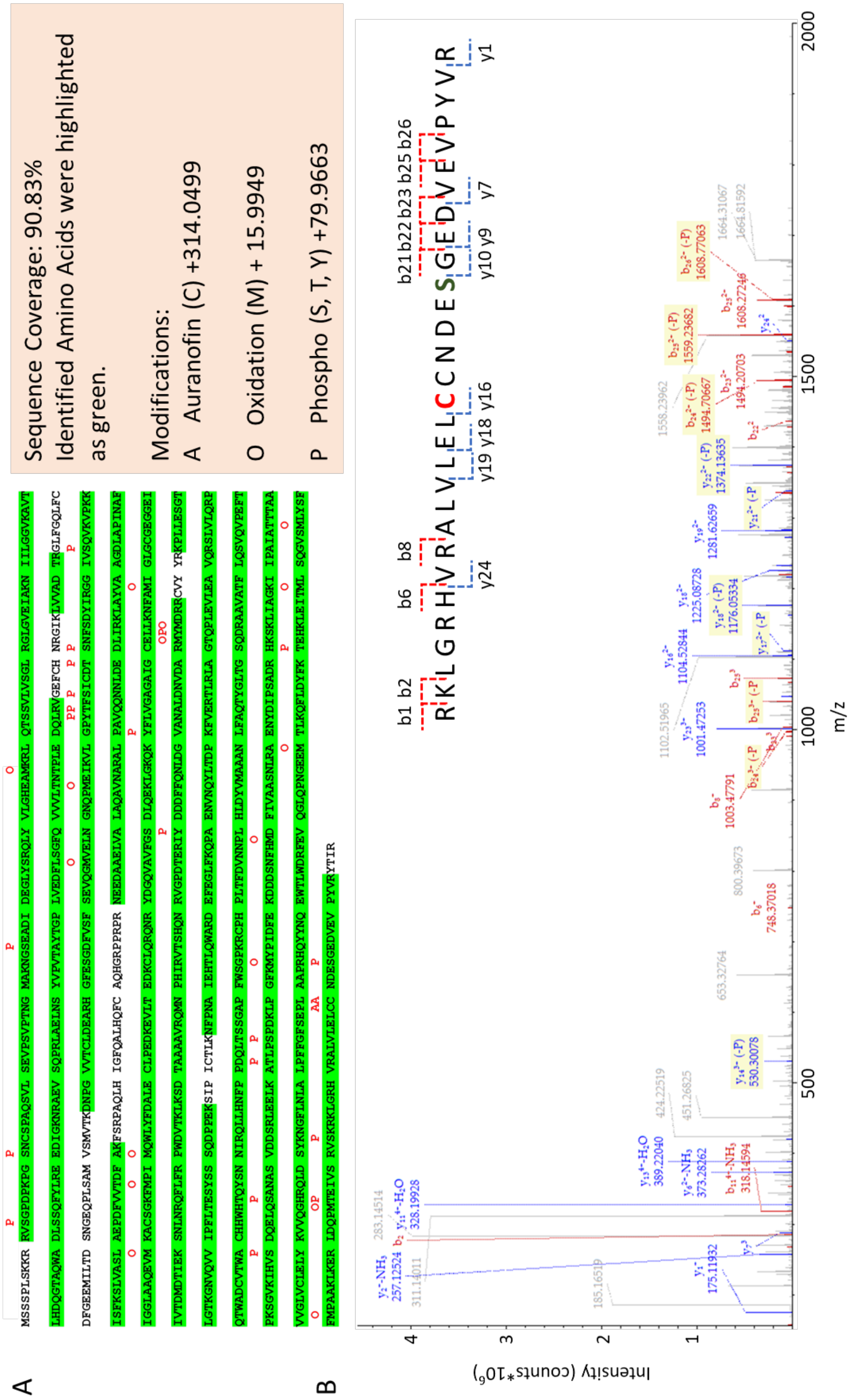

Suppl. Fig. 2: LC-MS/MS analysis for identification of the AF conjugations sites on UBA1. (a) Sequence coverage for the UBA1 protein with various modifications (A,O, P). **(b)** MS/MS spectra which matches to peptide sequence RKLGRHVRALVLELCCNDESGEDVEVPYVR contains AF conjugation site on C1039.

Suppl. Fig. 3

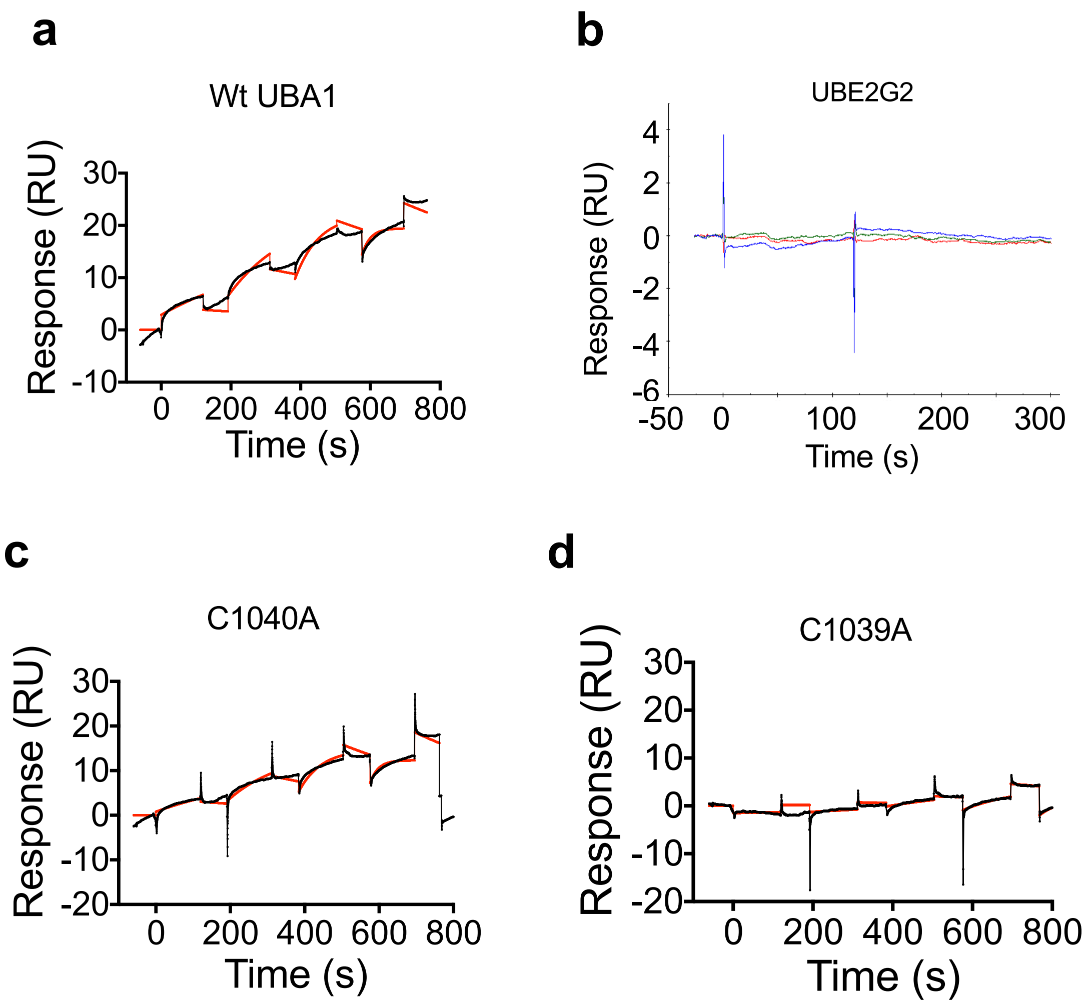

Suppl. Fig. 3: Sensorgrams of AF binding to UBA1 and its mutants (C1040A) and (C1039A) as well as UBE2G2.
Sensorgrams were obtained for 1.2, 3.7, 11.1, 33.3 or 100 nM AF against the indicated proteins immobilized on the CM5 sensor chip. UBA1 mutants were coupled to the surface of a Biacore CM5 sensor chips by direct immobilization. Ligand binding was performed at flow rate of 30 μL/ min in HBS running buffer. The association was recorded by Biacore. The assay was performed under condition that the binding surface was regenerated. The black line is signal line, and the red line is fit line. Source data are provided as a Source Data file.

Suppl. Fig. 4

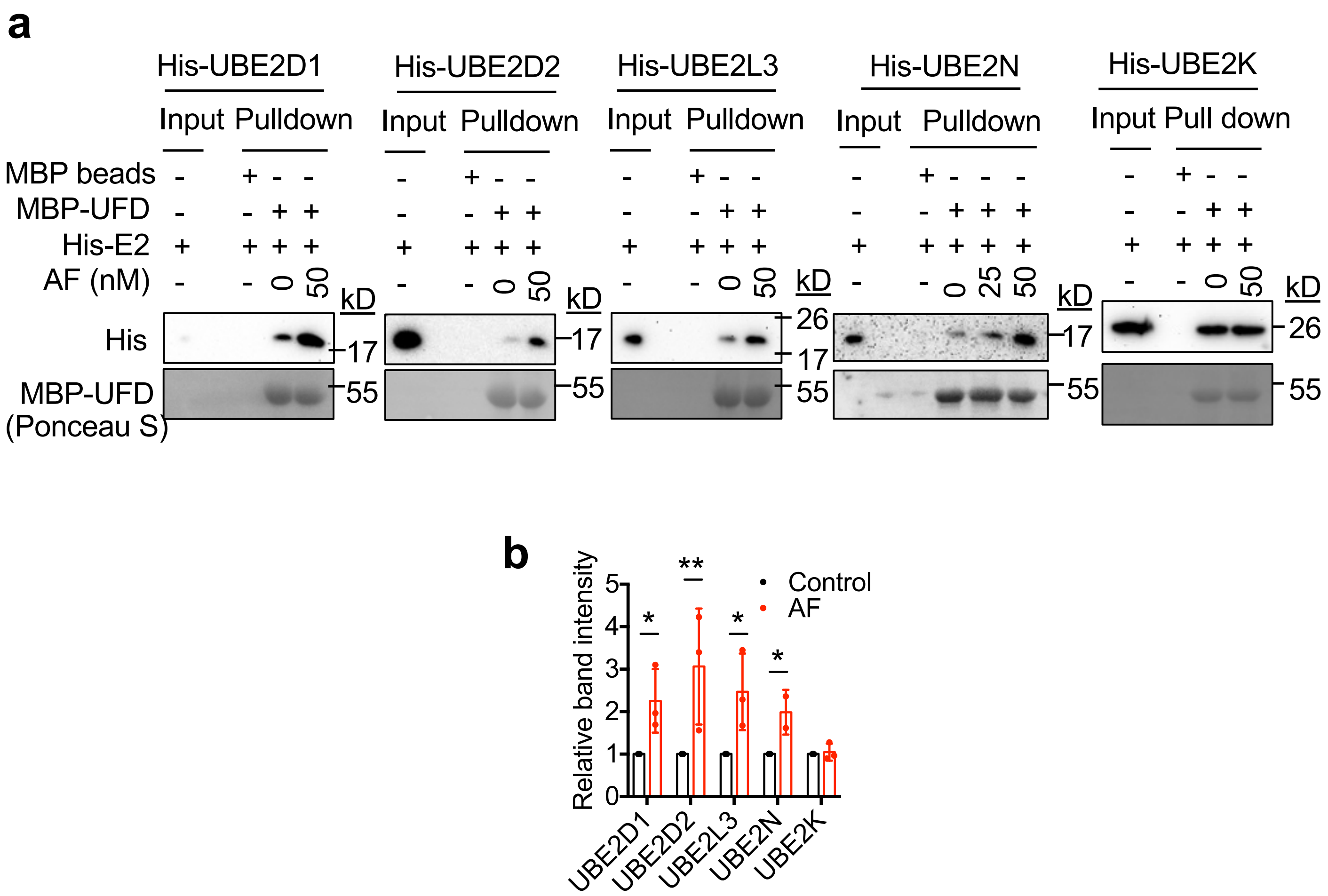

Suppl. Fig. 4: (a) AF enhances MBP-UFD interactions with E2s in *in vitro* MBP pulldown assays. All E2s were tagged with 6His and detected by anti-6His IB. MBP-UFD recombinant protein was detected with ponceau S staining. The pulldown assays were performed as described in Fig. S1 **(b)** The relative band intensities of E2s in DMSO or 50 nM AF-treated samples as shown in (A) were expressed as mean +/- S.D., $n = 3$ independent experiments. * $p < 0.05$, ** $p < 0.01$. p = 0.044, p = 0.0028, p = 0.0479, and p = 0.0378 for UBE2D1, UBE2D2, UBE2L3 and UBE2N, respectively. p values were calculated by two-tailed unpaired t-test. Source data are provided as a Source Data file.

Suppl. Fig. 5

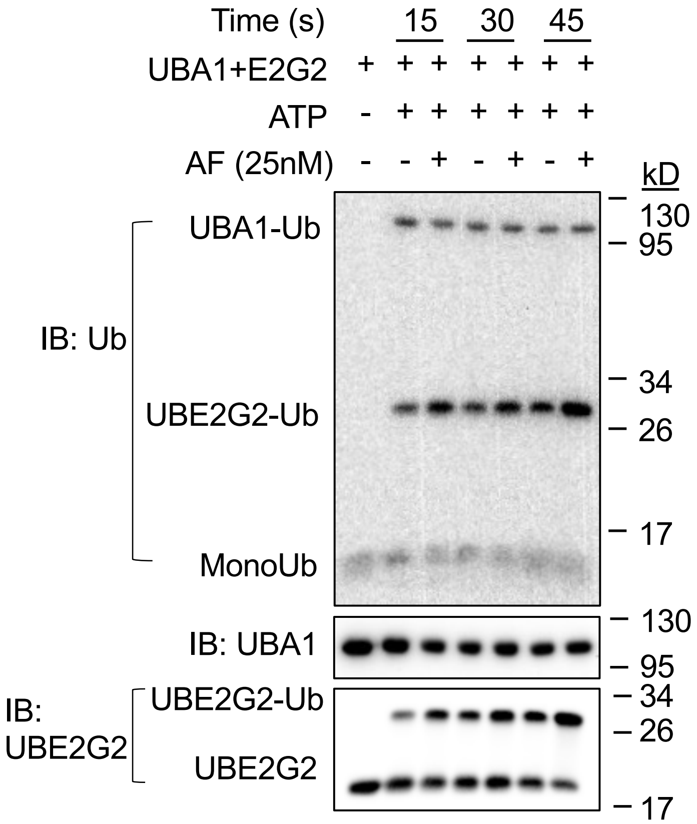

Suppl. Fig. 5: AF-induced time-dependent increases in ubiquitin charging to UBE2G2.

The assays were performed using purified recombinant proteins. 250 nM UBA1 alone or in combination with 4 µM His-UBE2G2 were treated with 25 nM AF. ATP (50 µM) was added to initiate the reaction. The reactions proceeded at 15 °C for 15, 30, 45 s, and stopped by adding non-reducing loading buffer followed by analysis by IB. Source data are provided as a Source Data file.

Suppl. Fig. 6

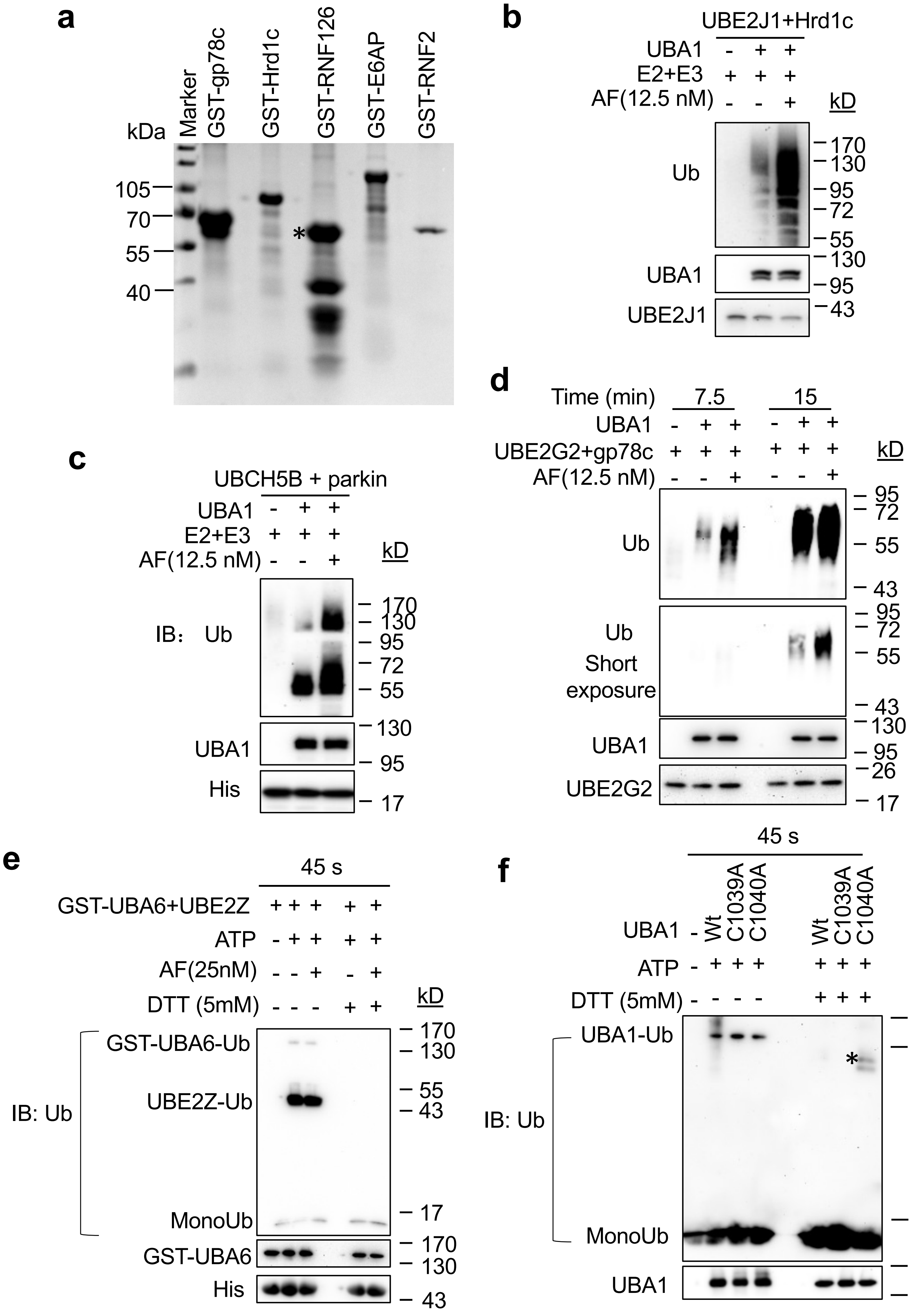

a
Marker
GST-gp78c
GST-Hrd1c
GST-RNF126
GST-E6AP
GST-RNF2
kDa
105
70
55
40
*
b
UBE2J1+Hrd1c
UBA1 - + +
E2+E3 + + +
AF(12.5 nM) - - +
kD
Ub
170
130
95
72
55
UBA1
130
95
UBE2J1
43
c
UBCH5B + parkin
UBA1 - + +
E2+E3 + + +
AF(12.5 nM) - - +
kD
IB: Ub
170
130
95
72
55
UBA1
130
95
His
17
d
Time (min)
7.5
15
UBA1 - + + - + +
UBE2G2+gp78c + + + + + +
AF(12.5 nM) - - + - - +
kD
Ub
95
72
55
43
Ub
Short
exposure
95
72
55
43
UBA1
130
95
UBE2G2
26
17
e
45 s
GST-UBA6+UBE2Z + + + + +
ATP - + + + +
AF(25nM) - - + - +
DTT (5mM) - - - + +
kD
IB: Ub
GST-UBA6-Ub
UBE2Z-Ub
MonoUb
170
130
55
43
17
GST-UBA6
170
130
His
43
f
45 s
UBA1 - Wt C1039A C1040A Wt C1039A C1040A
ATP - + + + + + +
DTT (5mM) - - - - + + +
kD
IB: Ub
UBA1-Ub
MonoUb
*
130
95
17
UBA1
130
95

Suppl. Fig. 6: Effects of AF on E3 activities and ubiquitin charging to UBA1 and its mutants *in vitro.*

(a) Recombinant E3s or E3 fragments used in in vitro ubiquitination described in Fig. 5A and S6B. The proteins were stained by coomassie blue. **(b-d)** Effects of AF on Hrd1c, Parkin, and gp78c-mediated ubiquitination *in vitro*. **(e-f)** Ubiquitin charging to E2s in vitro. The assays were performed using the protocol described in Fig. S5. Source data are provided as a Source Data file.

Suppl. Fig. 7

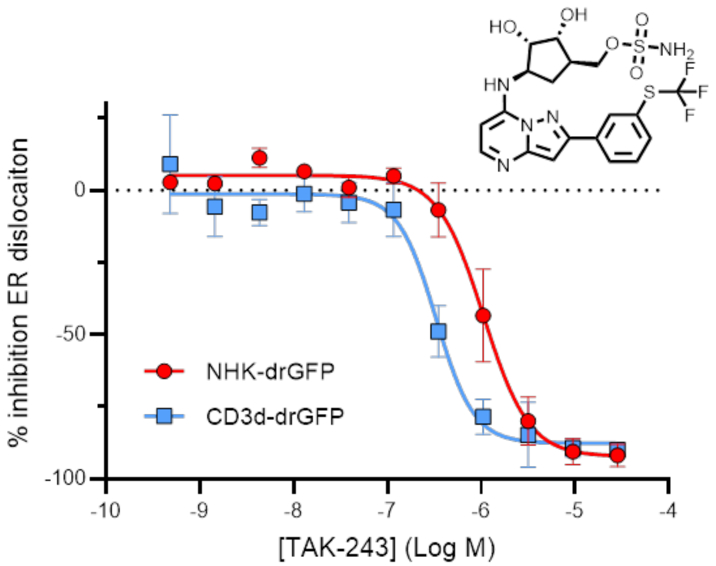

Suppl. Fig. 7: Inhibition of UBA1 activity by TAK-243 diminishes dislocation of NHK and CD3delta in HeLa cells.
Dislocation assays were performed in presence or absence of increasing amounts of TAK-243. Data are presented as mean values +/- S.D., *n* = 3 biologically independent samples. Source data are provided as a Source Data file.

Suppl. Fig. 8

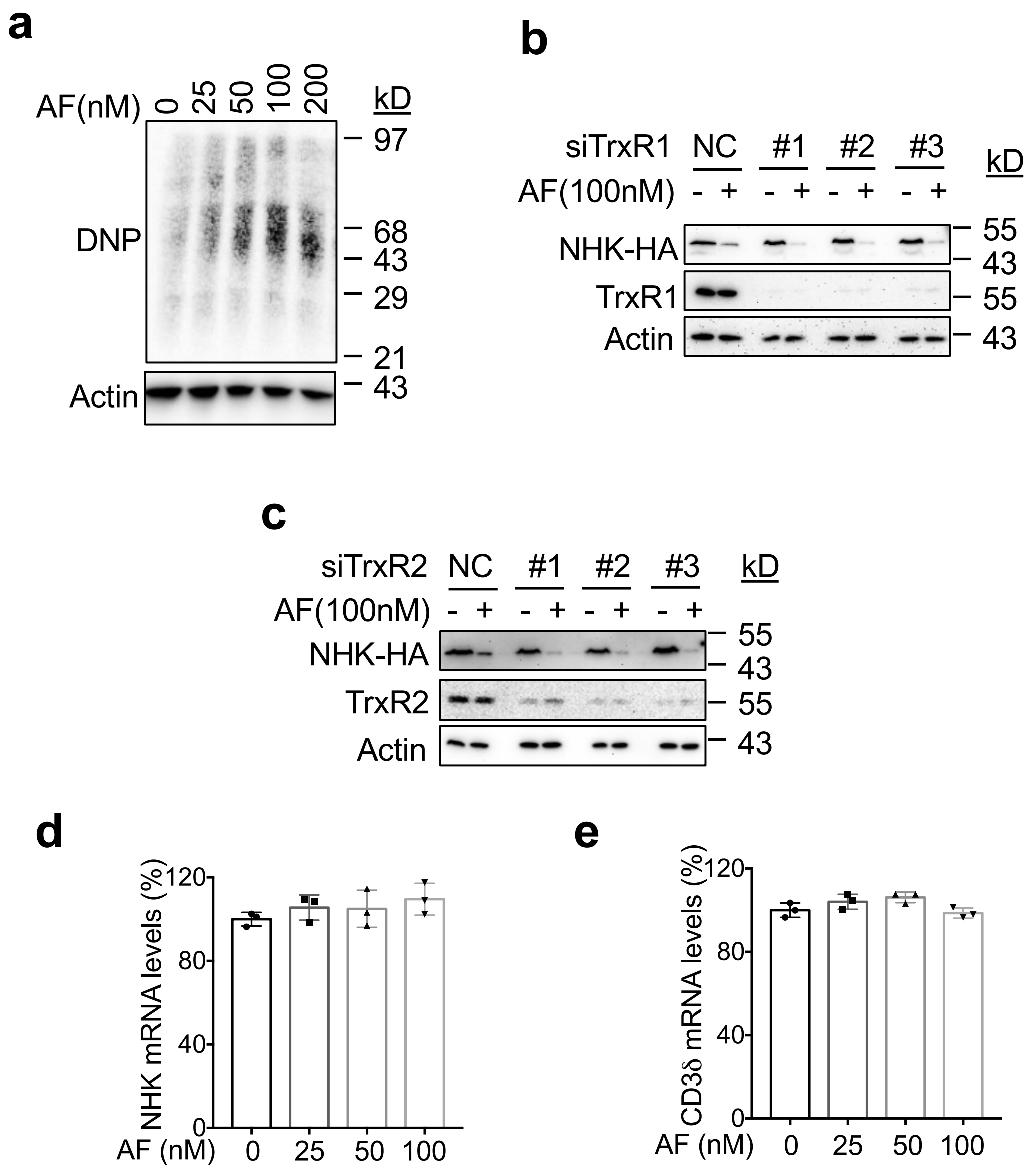

Suppl. Fig. 8: (a) AF induces protein oxidation as revealed by OxyBlot. OxyBlot detect carbonyl groups introduced into proteins by oxidative reactions. HeLa cells treated as indicated were processed for blotting using OxyBlot (S7150, OxyBlot Protein Oxidation Detection Kit, Millipore Sigma) following the manufacturer's instruction. DNP: 1-3 dinitrophenylhydrazone. **(b, c)** Knockdown of TrxR1 (b) or TrxR2 (C) does not affect AF-induced NHK degradation. **(d, e)** NHK and CD3delta mRNA levels were quantified by quantitative RT-PCR. The assays were performed using total RNA extracted from cells used in Fig. 5d and h. Data are presented as mean values +/- S.D., *n* = 3 biologically independent samples. Source data are provided as a Source Data file.

Table S1. Primers

Constructs	Primer sequence
pCIneo-HA-UBA1	CTTTGCTAGCCACCATGGCTTACCCATACGATGTTCCAGATTACGCTATGTCC AGCTCGCCGCTGTC
C1039A	F: CTGGTGCTTGAGCTcgcCTGTAACGACGAG<br>R: CTCGTCGTTACAGgcgAGCTCAAGCACCAG
C1040A	F: CTGGTGCTTGAGCTcTGCgcTAACGACGAGAGC<br>R: GCTCTCGTCGTTAgcGCAgAGCTCAAGCACCAG
E1037A	F: CGCTGGTGCTTGCGCTGTGCTGTAAC<br>R: GTTACAGCACAGCGCAAGCACCAGCG
E1049A	F: GCGAGGATGTCGcGGTTCCCTATGTC<br>R: GACATAGGGAACCgCGACATCCTCGC

Table S2. Oligos used in genome editing

UBA1 C1039A ssODN donor	TGACAGAGATTGTGAGCCGTGTGTCGAAGCGAAAGCTGGGCC GCCAtGTaaGaGCaCTaGTcCTTGAGCTGgctTGTAACGAtGAatcCG GaGAaGAcGTgGAGGTTCCCTATGTCCGATACACCATCCGCTGA CCCCGTCTGCTCC	
sgRNA 1	Forward	cacc GCACCAGCGCCCGCACGTGG
	Reverse	aaac CCACGTGCGGGCGCTGGTGC
sgRNA 2	Forward	caccg CGAGAGCGGCGAGGATGTCG
	Reverse	aaac CGACATCCTCGCCGCTCTCGc
PCR screening	Forward	GCAGGCGATGTGGGAGTTAT
	Reverse	GGAGCCGTTCTTTGCTAGC
PCR for DNA sequencing	Forward	GCAGGCGATGTGGGAGTTAT
	Reverse	CTCCGGATTCATCGTTACAAGC
